# *Ipomea hederacea* Jacq.: A Medicinal Herb with Promising Health Benefits

**DOI:** 10.3390/molecules171113132

**Published:** 2012-11-05

**Authors:** Muhammad Zia-Ul-Haq, Muhammad Riaz, Vincenzo De Feo

**Affiliations:** 1 Department of Pharmacognosy, University of Karachi, Karachi-75270, Pakistan; Email: ahirzia@gmail.com (M.Z.-U.-H.); pharmariaz@gmail.com (M.R.); 2 Department of Pharmaceutical and Biomedical Sciences, University of Salerno, Salerno 84100, Italy

**Keywords:** *Ipomea hederacea*, phytochemistry, pharmacology, compositional studies

## Abstract

*Ipomea hederacea* Jacq. (kaladana or ivy leaf morning-glory), a member of the family *Convolvulaceae*, is used primarily for its seeds and recognized for its medicinal properties, especially in Asian countries. This medicinal herb contains various valuable chemical constituents such as ecdysteriods, steroidal glycosides, aromatic acids, triterpenes, amino acids, organic acids, mineral elements and vitamins. A number of pharmacological properties such as diuretic, anthelmintic, blood purifier, deobstruent, laxative, carminative and anti-inflammatory actions have been ascribed to this plant, besides its use to treat abdominal diseases, fevers, headache and bronchitis. This review focuses on compositional, medicinal and therapeutic properties of this plant, as a potential sources of bioactive molecules for medicinal and nutraceutical applications.

## 1. Introduction

**Figure 1:**
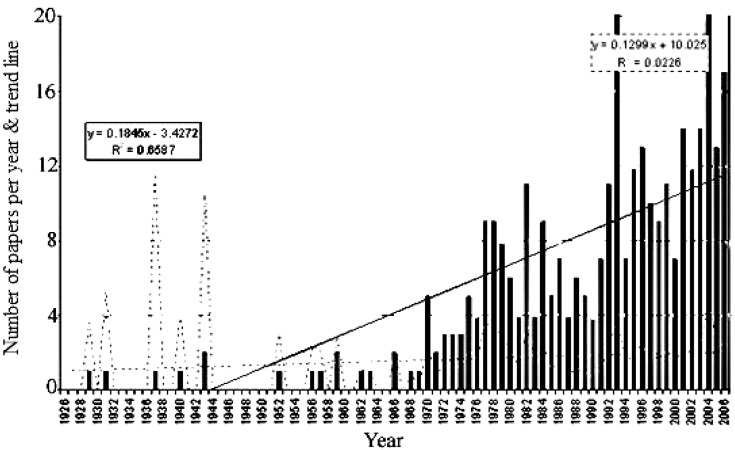
Popularity of *Ipomea hederacea* over time [11].

Plants are old friends of humans who rely on plants directly or indirectly for food, shelter, aesthetic purposes and for the treatment of diseases. Plants can considered as biological factories for the production of various medicinal compounds. Hence plants enjoy the attraction of common man as well as of the scientific community for investigation, authentication and rationalization of their food and therapeutic effects. As a part of our continuous efforts to explore the medicinal flora of Pakistan [1,2,3] we focused on a member of the family *Convolvulaceae* that comprises nearly 1,650 predominantly tropical species. The genus *Ipomoea*, with approximately 500–600 species, is the largest in the family [4] and twenty species are found in Pakistan [5]. Plants of this genus have been in continuous use since immemorial time for different nutritional, medicinal, ritual and agricultural purposes. Various species of *Ipomoea* have been extensively used in local traditional medicine in many countries for the treatment of several diseases [6]. *Ipomoea hederacea* Jacq. is known in the flora of Pakistan not only for its medicinal and therapeutic attributes (Figure 1), but it is frequently grown in gardens for its ornamental flowers and often runs wild in hedges and wastelands [7,8,9,10]. In light of its phytochemical, medicinal and therapeutic importance, this review focuses on its botanical description, traditional uses, phytochemical studies, toxicological and pharmacological aspects.

## 2. Botanical Description

### 2.1. Scientific Name

*Ipomoea hederacea* Jacq.: *Ipomoea*: from Greek *Ips* which means “a worm,” and *homoios* which means “like,” thus *Ipomoea* means “like a worm”, referring to the twining habit of the plant’s growth; *hederacea*: of or pertaining to ivy [12].

### 2.2. Common Names

Ivy leaf morning-glory, Woolly morning-glory (as flowers wilt after one day).

### 2.3. Local Names in Pakistan

Habbun-nil (due to the blue color of its corolla *nil* is used for blue color in Pakistan); Kaladana (due to the black color of the seeds *kala* means black and *dana* means seed).

### 2.4. Habitat and Distribution

Habitats include abandoned fields, areas along roadsides and rail*roads*, gardens and miscellaneous waste areas. *I. hederacea* is frost intolerant and is commonly found in warmer climates. This species frequently occurs in flower beds and cultivated areas. Because this species is now found throughout the World there is some confusion about its origin. It seems the this species orginates from the American tropics and it pertains to the flora of the south east of United States [5,12,13]. It is also distributed in Pakistan, Kashmir and India [14,15,16].

### 2.5. Plant Description (Figure 2)

Ivy-leaved morning-glory is an annual trailing vine (6–8 feet tall), branching occasionally and flowering from July to September. The preference is full or partial sun, mesic conditions, and fertile soil. The seeds don’t germinate until the soil becomes warm during early summer. This plant can be aggressive and it is quite common in disturbed areas, especially cultivated fields, where it can be a major pest. This is both an attractive vine and common weed of agronomic, horticultural, and nursery crops, and can be tough to get rid of once established [17,18]. The 2 inch (5 cm) wide, funnel-shaped flowers open only in the morning and have five hairy sepals. The bases of the flowers are densely hairy. The flowers are pollinated by bumblebees and other long-tongued bees. These are generally blue, but can show traces of purple, magenta, or white. The flowering stalks develop from the axils of the leaves and are quite short (¼" or less), producing 1–3 flowers. The flowers are about 2" across and bloom primarily during the mornings on sunny days. The throat of the corolla is white, where the reproductive organs of the flower form a white column with a knobby tip. The hairy green calyx is divided into five lobes that are linear-lanceolate and about ¾" long. These lobes often curl outward at their tips. Each flower is replaced by a 3-celled rounded capsule containing 4–6 seeds. The rather large seeds are brown to black and wedge-shaped. They have a dull surface [19,20]. The leaves are alternate, and indented at the base. They are three-lobed but they are quite variable and can also be five-lobed or heart-shaped, even on the same plant. Each lobe is widest in the middle and tapers to a blunt tip. The margins of the leaves are smooth and somewhat undulating, while the upper surface is more or less hairy. The petioles are hairy and almost as long as the leaves. The leaves are usually up to 4" long and are hairy [21,22]. The round stems are light green to dull red, and more or less covered with white hair. Stem twines about surrounding vegetation with their apex in a dextral fashion, or sprawl about haphazardly or more commonly climbing, reaching 10 feet in length. Stems also have hairs that are erect. The root system consists of a taproot. This plant spreads by reseeding itself [23,24]. Cotyledons are notched at the apex, and this notch forms an angle between the lobes that is less than 90 degrees. The cotyledons are only shallowly or moderately indented, and the lobes are usually rounded or only slightly pointed. Cotyledons are also notched at the base, and are close to square in outline, with only a slight flare outwards. The first true leaf is unlobed [25,26]. The seeds resemble in shape the “quarter” of an orange, there being two flat sides meeting at an acute angle and an arched back. They are dull black, about 5 mm long and 3 mm wide, weight about 34 mg; the hilum is distinct as a brown, slightly hairy, depressed spot. A transverse section shows plaited cotyledons in which small, slightly darker resin cells may be seen. The taste is at first not marked, but is subsequently acrid [27,28,29]. 

**Figure 2:**
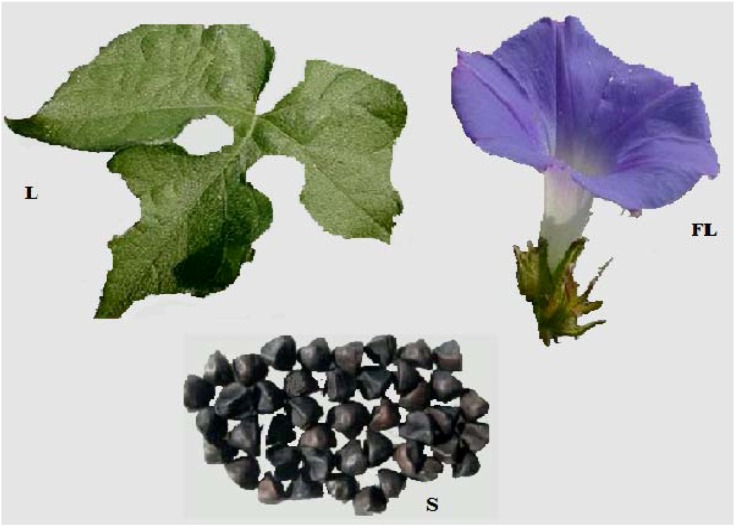
*Ipomea hederacea* Jacq., FL, flower; L, leaf, S, seed.

### 2.6. Traditional Uses

*I. hederacea* seeds and the resin extracted from them are used as medicines. The seeds have cathartic properties like those of jalap (*Ipomoea jalapa* (L.). Besides the resin, an extract, a tincture, and a compound powder have been introduced into the Pharmacopoeia of India. The resin, which has been introduced into medical practice in India under the name of “Pharbitisin”, has a nauseous, acrid taste and an unpleasant odor, especially when heated [30,31]. The seeds are regarded as diuretics, anthelmintics, aphrodisiacs, blood purifiers and deobstruents, and that they are prescribed in dropsy and constipation, to promote menstruation, and to produce abortion. They are claimed to be laxative, carminative, and useful in treatment of inflammations, abdominal diseases, fevers, headache and bronchitis. The juice of the leaves is used to treat eye inflammations, cataracts and films over the eyes; in the ear, the juice helps hearing, and cures ear noises. The herb is considered useful in oedema, ascites, fever, constipation, flatulence and epistaxis. Seeds are also useful in skin diseases like leucoderma and scabies, gout, cephalalgia, hepatopathy and splenopathy [32,33]. The seeds are rubbed on the male genitals to treat erectile dysfunction and on female genitals for lubrication purposes and to increase sexual desire. Paste of seeds is applied topically for cosmetic purposes as it removes dry skin and freckles. The topical application is also believed to be associated with weight loss.

## 3. Phytochemistry

*I. hederacea* contains a greater amount (15.78%) of crude resin than *I. muricata* Jacq (10.6%). Similarly the saponification number of the crude resin of *I. hederacea* was also greater (144.7) than that of *I. muricata* (116.7), while the acid numbers of both are fairly uniform [34]. Kathpalia and co-workers analyzed the seeds of *I. hederacea* and reported a fixed oil content of 9.38% with d_20_ = 0.918, n_20_ = 1.474, viscosity at 20° = 0.2938, saponification no. = 190.48, acetyl no. = 5.19, acid no. = 3.45, I. no. = 121.5, unsaponifiable = 1.98%; the mixed fat acids fraction had a neutralization value of 190.90, I no. 126.5 [35]. Seeds contain 4%–15% crude resinous matter; a fixed oil (12.4%) and small amount of saponins, mucilage and tannins [36]. Ahmad and co-workers confirmed the presence of saponins, tannins, terpenes, alkaloids and proteins in an ethanolic extract of seeds [37]. Alkaloids, reducing sugars, terpenoids, flavonoids, saponins and tannins were also reported [16]. The seed oil of *I. hederacea* was evaluated for color, refractive index, acid value, saponification value and unsaponifiable matter by standard IUPAC methods for the analysis of oils (Table 1) [38].

**Table 1:** Physico-chemical parameters of the seed oil of *I. hederacea*.

Parameter	Property/Content
Color	Light yellow
Refractive index	1.47
Specific gravity	0.92
Unsaponifiable matter	1.73
Acid value	2.98
Saponification value	190.48

**Figure 3:**
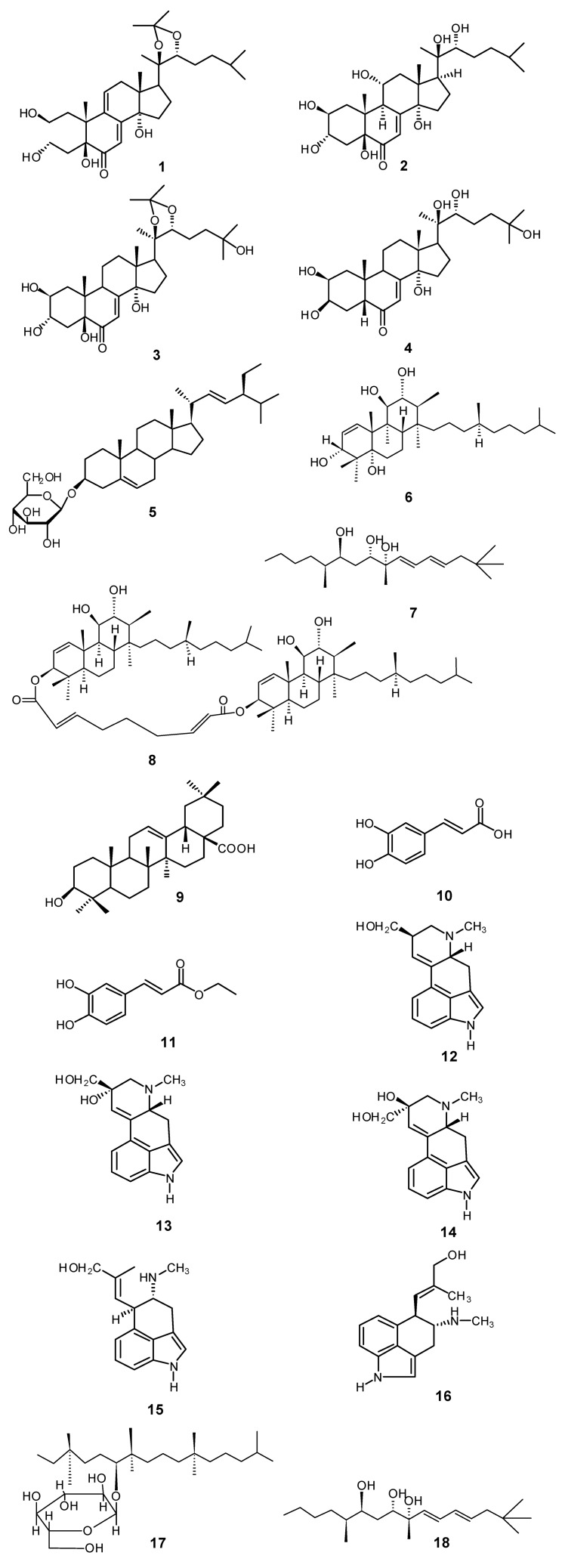
Phytochemicals isolated from *I. hederacea*: hederasterone A-20,22-monoacetonide (**1**), hedrasterone B (**2**), 20-hydroxyecdysone-20,22-monoacetonide (**3**), 20-hydroxyecdysone (**4**), stigmasterol-3-o-β-D-glucoside (**5**), hederaceterpenol (**6**), oleanolic acid (**7**), caffeic acid (**8**), ethyl caffeate (**9**), lysergol (**10**), penniclavine (**11**), hederacetriol (**12**), isopenniclavine (**13**), chanoclavine (**14**), hanoclavine (**15**), elymoclavine (**16**), hederaterpenoside (**17**), hederacyl triterpenoid (**18**).

Phytochemical studies on *I. hederacea* have been conducted since the mid 1960s and one of the early studies reported the isolation of the alkaloids lysergol, chanoclavine, penniclavine, isopeniclavine and elymoclavine from seeds. Chanoclavine, elymoclavine, lysergol and penniclavine were known to be psychoactive [36]. Isopenniclavine was reported to be psychotomimetic, while penniclavine, lysergol and elymoclavine were reported to be psychotropic [6]. Ahmad and co-workers reported the presence of tiglic acid, methyl ethyl acetic acid and α-methyl-β-oxybutyric acid in an ether soluble portion of a glycosidal fraction in the resin of *I. hederacea* seeds and the presence of pharbitinic acid, phytoecdysone, a plytosteratin, lysergol and chanoclavine [7]. Five ecdysteriods, two steroidal glycosides, one triterpene and two aromatic acids have also been isolated. Among ecdysteriods, hederasterone A, hederasterone A-20,22 monoacetonide and hedersterone B were reported for the first time from this plant [18]. Other reported compounds were 20-hydroxyecdysone, stigmasterol 3-O-β-D-glucoside, β-sitosterol-3-*O*-β-D-glucoside, oleanolic acid, caffeic acid, ethylcaffeate. In a butyryl-cholinesterase enzyme inhibitory assay, only hedersterone B exhibited inhibition and caffeic acid and ethylcaffeate showed antioxidant and lipoxygenase inhibition activity [18]. The most recent phytochemical investigation of the seeds of *I. hederacea* resulted in the isolation of hederaceterpenol, hederacetriol, hederaterpenoside, hederacyl triterpenoid and stigmast-5-en-3-*O*-β-D-glucopyranoside [7] (Figure 3). Compositional studies of *I. hederacea* seed and seed oil have been carried out by Kathpalia and Dutt [35] and later by Zia-Ul-Haq and co-workers [38]. (Table 2, Table 3, Table 4, Table 5, Table 6, Table 7). According to these studies, the proximate chemical composition (Table 2), the percent composition of amino acids in seeds (Table 3), their mineral content (Table 4), the percent composition of the oil (Table 5) the fatty acid (Table 6) and the tocopherol profiles (Table 7) indicated that the plant oil can be considered a substantial source of essential nutrients and may be used as food once its toxicity profile is established.

**Table 2:** Percent proximate composition of *I. hederacea* seeds.

Component	Content
Crude protein	23.36
Total lipids	14.09
Total carbohydrates	37.06
Crude fiber	16.55
Moisture	5.29
Ash	3.65

**Table 3:** Percent composition of amino acids in *I. hederacea* seeds.

Amino acid	Content
Isoleucine	5.03
Leucine	6.59
Lysine	4.25
Methionine	1.17
Phenylaniline	6.24
Threonine	3.07
Tryptophan	1.88
Valine	7.10
Arginine	5.50
Histidine	3.55
Alanine	3.99
Aspartic acid	10.82
Cystine	0.90
Glutamic acid	22.71
Glycine	5. 36
Proline	4.46
Serine	4.02
Tyrosine	2.58

**Table 4:** Mineral content (mg/100 g) of *I. hederacea* seeds.

Metals	Content
Calcium	317.41
Copper	4.62
Iron	9.85
Magnesium	179.14
Manganese	6.37
Phosphorus	596.19
Potassium	978.46
Sodium	106.32
Zinc	4.01
Na:K	0.11
Ca:P	0.53

**Table 5:** Percent composition of *I. hederacea* seed oil.

Oil class	Composition
Hydrocarbons + waxes	0.40
Steryl esters	0.60
Triacylglycerols	80.20
Free fatty acid	1.20
Diglycerols	0.70
Monoglycerols	1.00
Polar lipids	6.50
Phospholipids	4.80
Unidentified	4.60

**Table 6:** Fatty acid profile (%) of *I. hederacea* seed oil.

Fatty acid	*Ipomoea hederacea* [38]	*Ipomoea hederacea* [35]
Palmitic acid (16:0)	17.03	5.93
Palmitoleic acid (16:1)	1.10	-
Stearic acid (18:0)	6.00	20.37
Oleic acid (18:1)	19.50	43.98
Linoleic acid (18:2)	52.09	14.54
α-Linolenic acid (18:3)	4.28	5.99
Arachidic acid (20:0)	-	7.79
Eicosaenoic acid (20:1)	-	-
Behenic acid	-	1.29

**Table 7:** Tocopherol profile (mg/100g) of *I. hederacea* seed oil.

Tocopherol	*Ipomoea hederacea*
α-Tocopherol	0.50
β-Tocopherol	1.60
γ-Tocopherol	28.70
δ-Tocopherol	2.30
Total	33.10

Various phenols like erulic acid, coumaric acid-hexoside, ferulic acid-hexoside and sinapic acid-hexoside were tentatively identified in *I. hederacea* seeds, based on their mass spectral characteristics [38].

## 4. Pharmacological activities of *I. hederacea*

### 4.1. Antioxidant Activity

The antioxidant activity of different extracts of *I. hederacea* was evaluated by Rehman and co-workers by four methods, 1,1-diphenyl-2-picrylhydrazyl (DPPH) free radical scavenging activity, ferric reducing antioxidant power (FRAP) assay, total antioxidant activity and Folin-Ciocalteau reagent assay for the determination of total phenolics. It was reported that an ethyl acetate soluble fraction showed the highest value of percent inhibition of DPPH (83.26%) at a concentration of 125 µg/mL, with an IC_50_ of 60.28 µg/mL. This fraction also displayed the highest FRAP value (80 µg TE/mL), the higher total antioxidant activity (0.25 ± 0.31) as well as highest total phenolic contents (62.35 µg GAE/g) as compared to other fractions [16]. Some extracts of *I. hederacea* showed good antioxidant capacity, reducing different types of radicals [16]. The differences found among the previous and recent studies (Table 8) were explained in terms of difference in genotype, growing condition, agronomic practices employed, season, maturity, post-harvest storage and processing conditions and solvent used for extraction [38]. 

**Table 8:** Antioxidant activity of *I. hederacea*.

Antioxidant Assay	Content
Total phenol content (mg CAE/g)	14.33
TEAC (µmol TE/g)	93.57
FRAP (µmol Fe++/g)	278.24
TRAP (µmol TE/g)	69.02

### 4.2. Hepatoprotective Activity

An ethanolic extract of the plant showed significant hepatoprotection in carbon tetrachloride treated rats [39].

### 4.3. Antibacterial Activity

The disk diffusion assay was employed in the study by Zia-Ul-Haq and co-workers to investigate the antibacterial potency of *I. hederacea*. The bacterial strains studied included *Escherichia coli*, *Citrobacter* sp., *Staphylococcus aureus*, *Pseudomonas aeruginosa*, *Salmonella typhi*, *Micrococcus luteus*, *Proteus mirabilis* and *Bacillus subtilis*. The resultsindicated that *I. hederacea* had potent activity against all the tested microorganisms, being *B. subtilis* the most sensitive strain [40]. Singh and co-workers [41] also reported antimicrobial properties of various extracts of the plant. A methanol extracts of seeds of *I. hederacea* exhibited antibacterial activity, with minimum inhibitory concentration (MIC) of 2 mg/mL against *Escherichia coli* and *Pseudomonas aeruginosa*. The methanol extract was found to be more active than the other extracts.

### 4.4. Antifungal Activity

The antifungal activity of *I. hederacea* extracts was evaluated against nine fungal strains, viz., *Aspergillus parasiticus*, *A. niger*, *A. effusus*, *Yersinia aldovae*, *Candida albicans*, *Fusarium solani*, *Macrophomina phaseolina*, *Saccharomyces cerevisiae* and *Trichophyton rubrum* by the disk diffusion assay. The extracts showed significant activity against these strains [40]. The antifungal activity of was also performed against *Alternaria brassica*, *A. braceacola* and *Aspergillus niger*. The methanol and chloroform extracts showed moderated as well as significant activity against the strains [41].

### 4.5. Nematicidal Activity

An ethanolic extract of the seeds of *I. hederacea* was evaluated for nematicidal activity against larvae of *Meloidogyne incognita*, *Meloidogyne javanica* and *Cephelobus litoralis.* Moderate time- and concentration-dependent activity was reported [42].

### 4.6. Insecticidal Activity

Different doses of a crude methanol extract of the plant showed insecticidal activities against *Tribolium castaneum*. At the dose of 0.01 to 300 mg/kg mortality was 100%, while at 0.001 mg/kg no mortality was observed; all these results are time dependant [37].

### 4.7. Cytotoxic Activity

In order to discover novel antitumor agents, Nam and Lee approximately screened 180 methanol extracts of medicinal plants for their cytotoxicity in cultured human lung (A549) and colon (Col 2) cancer cells. As a result, 17 natural product extracts were found to be active in the criteria of IC_50_ < 20 μg/mL. *I. hederacea* also showed cytotoxic potential [43].

### 4.8. Analgesic Activity

Analgesic activity was investigated using Writhing test and hot plate method. *I. hederacea* extract inhibited acetic acid induced writhing in mice and increased the pain threshold according to the hot plate method. The methanol extract showed a promising analgesic action at lower doses. Analgesic effect was significant at 1, 0.025 and 0.0125 mg/kg doses [37].

### 4.9. Toxicological Studies

The result of oral toxicity of a crude methanol extract of *I. hederacea* was observed in mice at a dose range of 0.0125–300 mg/kg. The start of lethal effects was recorded at the dose of 300 mg/kg. Oral acute toxicity indicated that there was 50% mortality at higher doses. Dose dependent toxic effects in mice behaviour included convulsions, tremors, unsteady gait and respiratory distress to death. The LD_50_ value was found 229.2 mg/kg, while the ED_50_ was found to be 0.0125–1 mg/kg [37].

### 4.10. Other Activities

A crude methanol extract of *I. hederacea* at low dose showed a promising CNS stimulant activity and potential towards improvement of muscles activity. Neuropharmacological activities were significant at 1, 0.025 and 0.0125 mg/kg doses [37].

## 5. *Ipomea hederacea* in Patents

An important health food for treating rheumatoid arthritis, degenerative arthritis and osteoporosis was developed and the major part of this formulation was constituted by *I. hederacea* [44]. The plant is also a part of a skin-whitening composition along with several other medicinal plants, this composition being recommended for treating skin diseases such as fleck on the face [45].

## 6. Conclusions

*Ipomoea hederacea* is a medicinal herb currently gaining popularity among researchers due to its potential health benefits. The information presented in this review shows the potential nutritional importance of this plant and its role in improved nutrition and health. It is an affordable source of protein, carbohydrates, minerals and vitamins and health-promoting fatty acids. However *in vivo* toxicological studies should be performed before inclusion of its seeds in foods. The survey of the literature revealed the presence of alkaloids and triterpenoids. The plant may be exploited as a source for seed gums and may serve as a renewable reservoir of industrial gums. It has also revealed a broad spectrum of pharmacological activities. More advanced techniques should be used to further explore high-value bioactive constituents responsible for tagged bioactivities.
